# Elevated cortisol during play is associated with age and social engagement in children with autism

**DOI:** 10.1186/2040-2392-1-13

**Published:** 2010-09-27

**Authors:** Blythe A Corbett, Clayton W Schupp, David Simon, Niles Ryan, Sally Mendoza

**Affiliations:** 1 Department of Psychiatry, Vanderbilt University, 1601 23rd Avenue South, Nashville, TN 37212, USA; 2 Vanderbilt Kennedy Center, PMB 40, 230 Appleton Place, Nashville, TN 37203, USA; 3 Graduate Group in Epidemiology, University of California, Davis, One Shields Avenue, Davis, CA, 95616, USA; 4 Department of Psychiatry and Behavioral Sciences, University of California, Davis, 2230 Stockton Blvd., Sacramento, CA, 95817, USA; 5 The M.I.N.D. Institute, University of California, Davis, 2825 50th Street, Sacramento, CA, 95817, USA; 6 Department of Psychology, University of California, Davis, 134 Young Hall, Davis, CA, 95616, USA

## Abstract

**Background:**

The hallmark characteristic of autism is impaired reciprocal social interaction. While children find social interaction stress-reducing, many children with autism may find social interaction stress-inducing. The current study was designed to examine stress responsivity as measured by cortisol by comparing children with autism to neurotypical peers during an ecologically valid 20-minute playground paradigm.

**Methods:**

The experiment involved sets of three children: a child with autism, a neurotypical child, and a confederate. Participants included 45 prepubescent males between 8 and 12 years of age (21 with autism and 24 neurotypical children).

**Results:**

Children with autism showed fewer initiations (χ²(1) = 4.03, *P *= 0.044), rejected initiations from others more (χ²(1) = 7.10, *P *= 0.008) and spent less time interacting during motor (F(1,43) = 16.7, *P *= 0.0002) and cooperative (F(1,43) = 14.78, *P *= 0.0004) play. Repeated measures analysis of the cortisol values revealed a significant model (χ²(4) = 22.76, *P *< 0.0005) that included time of measurement, diagnosis and age as main effects and an interaction between diagnosis and age. Thus, as age increased among children with autism, they experienced enhanced cortisol levels while age did not modify expected cortisol levels for typical children. Stress responsivity was associated with more peripheral equipment play for motor (χ²(3) = 12.3, *P *= 0.006) and cooperative (χ²(3) = 8.24, *P *= 0.04) play as well as reduced nonverbal social skills during motor (χ²(1) = 5.52, *P *= 0.018) and cooperative play (χ²(1) = 4.53, *P *= 0.033).

**Conclusions:**

Overall, children with autism engaged in fewer social overtures and spent less time interacting than typically developing peers during play. The peer interaction paradigm resulted in significantly higher levels of cortisol in many children with autism. Distinct patterns emerged within the autism group based on developmental (older), biological (cortisol responder) and behavioral patterns (peripheral group interaction). The enhanced cortisol response was observed in children who voluntarily engaged in interaction; thus, it does not support the notion of a response to social threat. Rather, it appears to reflect attendant metabolic preparedness and enhanced arousal from engaging socially. The data suggest that many children with autism activate hypothalamic-pituitary-adrenal responses in relatively benign social situations, which appears to be a function of age and level of social engagement. The findings support the need to teach coping strategies in addition to fundamental social skills to youth with autism.

## Background

Autism is characterized by impairment in verbal and nonverbal communication, reciprocal social interaction and a restricted repertoire of activities and interests [1]. The symptoms fall on a continuum of severity referred to as autism spectrum disorder (ASD), in which impaired social functioning is the hallmark feature across the spectrum. Adequate social knowledge relies on the ability to interpret another person's behavior, to interact in both complex social groups and close relationships, to empathize and to predict how others will feel, think and act.

Play is critical for the development of social, cognitive and motor skills [2,3]. Even though poor reciprocal social interaction is the hallmark deficit in autism, surprisingly few observational studies of play exist [4-7] and they primarily utilize only questionnaires or contrived laboratory conditions. The type of environment and context can affect social interaction, with enclosed environments facilitating imitation and gross motor play in autism [8,9]. Playground observation can assist in screening for ASD [6] and may serve as an ecologically valid approach to elucidate social and psychobiological profiles in autism, including social stress.

Stress and anxiety are technically different constructs, and both have been reported from the earliest conceptualizations of autism [10] and alluded to in classification systems [11,12]. In this study, stress refers to the response to perceived threat to the physiological or psychological integrity of an organism, often leading to the increased release of glucocorticoids (e.g., cortisol) [e.g., 13, 14]. Anxiety, on the other hand, pertains to a feeling of apprehension or worry that may be state- or trait-based (e.g., [15]). Many children with autism have significant anxiety [16,17], and notable physiological stress has been reported [18-20]. Furthermore, these constructs can be closely linked and may co-occur.

Limited research has examined how stress may be associated with aspects of social functioning in autism [21-23]. While many children find social interaction stress-reducing, children with autism often appear to find social interaction stress-inducing. Increased stress and anxiety in autism may be the result of dysfunction of the amygdala, a brain structure involved in the detection of threats and mobilizing an appropriate behavioral response [24,25]. The amygdala is fundamentally involved in social cognition [26] and is a key regulator of the limbic hypothalamic pituitary adrenocortical (LHPA) axis and a mediator of processive stimuli [27].

The LHPA axis is highly regulated, and the system is dependent on the ability to maintain, respond and reset itself through a homeostatic process involving three primary interrelated processes: the maintenance of a diurnal rhythm, activation in response to stress or threat and the restoration of basal activity via negative feedback mechanisms. Once activated, a neuroendocrine cascade is initiated which results in the release of glucocorticoids from the adrenal gland. In humans, cortisol is the primary glucocorticoid, which, once released, results in a suite of metabolic changes and engages in a negative feedback loop to return the system to basal levels. Thus, cortisol is a widely used biological marker of both stress activation and restoration of homeostasis. Although the concentrations of cortisol in saliva are lower, it has been established that the correlation between plasma cortisol and salivary cortisol is high (0.71 to 0.96) and that the temporal changes of cortisol in saliva closely mimic those in blood in response to potentially stressful events [28,29]. Thus, salivary cortisol provides a useful, noninvasive biomarker for use with children.

The rhythmicity and responsivity of the LHPA axis in autism have been investigated, revealing abnormalities in neuroendocrine function, including an exaggerated stress response to various environmental events [18-20]. However, notable variability, exposure history and other factors may influence LHPA regulation and responsivity in autism [22,30,31]. Developmental research shows that stress reactivity is dynamic and that responsivity may vary based on context [32] as well as age and approach behavior in children [33]. Moreover, social variables can induce, enhance or diminish the stress response [34].

Despite the growing literature, research has been scant in terms of the influence of social factors, especially under more ecologically valid paradigms [22,35]. Autism is heterogeneous, and the social behavior that defines it is diverse [12]. It may be the case that differences in social behavior may also reflect distinct underlying psychobiological profiles related to LHPA responsivity [31].

The purpose of the investigation was to evaluate cortisol responsiveness in a naturalistic playground social setting. A well-established, direct observational measure of natural social behavior with peers in autism has been lacking. This led to the establishment of our peer interaction paradigm, which was developed with colleagues on the basis of transactional behavioral measures of social behavior in nonhuman primates [36-38], social initiation in autism [5] and clinical expertise in observational techniques in autism [39,40]. This ecologically valid design permits the careful investigation of social interaction in a play-based paradigm. It also allows real time assessment of social variables to be directly compared to stress reactivity.

In contrast to many studies of physiological responsivity which inherently aim to solicit a stress response [41], the paradigm was designed to emulate a "real life" playground to determine whether such environments would be deemed physiologically stressful. The peer interaction described below includes only three children and involves solicited but not forced interaction. There was no evaluative threat or peer rejection, both of which have been shown to activate the LHPA [41,42]. We reasoned that if a participant exhibited social stress under such benign conditions, then responses may be even more notable on a typical school playground with many children, enhanced stimulation and challenging social exchanges. On the basis of previous research showing enhanced stress responsivity in children with autism [18,22], we hypothesized that many children with autism would show an increase in salivary cortisol following the peer interaction compared to their average afternoon home level, baseline (arrival/acclimation) level and the cortisol values of neurotypical children. Our previous work led us to predict that variability in cortisol responsivity would be evident [31]. Thus, we hypothesized that social and biological phenotypes would be evident; specifically, enhanced social interaction would result in an increase in cortisol in the autism group, whereas more social interaction would be associated with reduced cortisol in the neurotypical group. Since it was a new paradigm, specific *a priori *hypotheses regarding the behavioral coding variables were not considered beyond the total social interaction time.

## Materials and methods

Informed written consent was obtained from parents, and verbal assent was obtained from all research participants prior to inclusion in the study. The Institutional Review Board of the University of California, Davis, approved the study.

### Participants

Participants included 45 prepubescent male children matched on age between 8 and 12 years, 21 children with high functioning autism and 24 typically developing children (three with autism had unusable data). The inclusion criteria for the experimental group consisted of boys diagnosed with autistic disorder (not Asperger syndrome or pervasive developmental disorder-not otherwise specified) based on DSM-IV criteria [1] and corroborated by standardized procedures (e.g., Autism Diagnostic Observation Schedule-Generic (ADOS-G [43])).

Children with autism were excluded if they demonstrated known co-occurring neurological disorders (e.g., seizures, *n *= 1) or genetic alterations (e.g., fragile X syndrome) or if they were unable to complete significant portions of the research protocol. The neurotypical participants had an absence of a neurodevelopmental disorder, learning disability or the presence of current or past psychiatric disorders determined by parent interview. The study also included age-matched confederates who underwent an assessment to rule out the presence of a disorder or disability. They were established in the MIND Institute subject tracking system and expressed interest in engaging in research. The confederates received the same compensation as the research participants and were scheduled based on the age of the autism/typical pair. Over the course of the study, three confederates were utilized and each received training from research personnel, observed other confederates prior to their initiation and received feedback following each playground interaction.

### Diagnostic and classification measures

The *Autism Diagnostic Observation Schedule *(ADOS [43]) is a semistructured interview designed to assess behaviors indicative of autism. For inclusion, a score of ≥10 on the social communication domain was required.

The *Wechsler Abbreviated Scale of Intelligence *(WASI [44]) is a measure of cognitive ability that was used to obtain an estimate of intellectual functioning. Participation required an IQ of ≥75.

The *Pubertal Development Scale *(PDS [45]) is a parent report measure which allows an estimate of the participant's level of pubertal development, an important consideration in developmental studies involving hormonal assays. We enrolled participants who had not formally entered puberty defined as having a score of 3 based on ratings of 1 (change not yet begun) in each of three categories: voice, pubic hair and facial hair.

### Dependent measures

The *Social Communication Questionnaire *(SCQ [46]) is a screening tool for ASD. Scores of ≥15 are suggestive of ASD, while scores of ≥22 are suggestive of autism. Neurotypical participants scoring ≥10 were excluded.

The *Social Responsiveness Scale *(SRS [47]) is a parent questionnaire addressing several domains of behavior characteristic of autism.

### Cortisol sampling protocol

**Figure 1:**
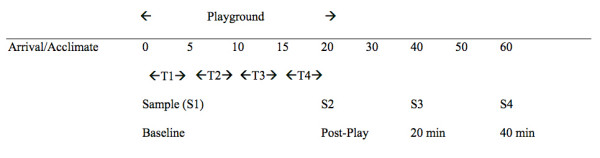
**Time line for cortisol sampling before and following peer interaction**. Figure T1 = Gross Motor Play, T2 = Solicited Motor Play, T3 = Cooperative Play, T4 = Solicited Cooperative Play. S1 = Baseline (Preplay), S2 = Postplay, S3 = 20 minutes Postplay, S4 = 40 minutes Postplay.

Our diurnal collection protocol is detailed elsewhere [48], and the data are part of a separate investigation. Briefly, basal levels of salivary cortisol were collected from the home four times per day (immediately upon waking, 30 minutes postwaking, in the afternoon and evening prior to bed) for six diurnal cycles. Morning and evening samples were collected prior to food consumption or brushing teeth. The afternoon samples were collected between 1300 and 1500 hours at least 1 hour after a meal, and the average afternoon sample was used as a comparison to baseline for the peer interaction stressor. The protocol was discontinued if the participant became ill (e.g., fever) and resumed once health status improved. For the stress protocol, four salivary samples were obtained, including (S1) a baseline sample taken after arrival (~15-minute acclimation) just prior to the playground peer interaction within a relaxed, private waiting room, (S2) postplay, (S3) 20 minutes postplay, and (S4) 40 minutes postplay (see Figure 1).

Our standardized collection procedures are detailed elsewhere [18,48]. In brief, the participant is given Trident Original Sugarless chewing gum (Cadbury Adams USA LLC, Parsippany, NJ, USA), which serves as a saliva stimulant, then the child deposits saliva into a tube by passive drool. Samples were stored in a -20°C freezer. Prior to assay, samples were thawed and centrifuged at 6000 rpm for 10 minutes to separate aqueous component from mucins and other suspended particles. Assays were performed using coated-tube radioimmunoassay kits (Siemens Medical Solutions Diagnostics, Los Angeles, CA, USA). Assay procedures were modified to accommodate overall lower levels of cortisol in human saliva relative to plasma as follows: (1) standards were diluted to concentrations ranging from 2.76 to 345 nmol/L, (2) sample volume was increased to 200 μl, and (3) incubation times were extended to 3 hours. Serial dilution of samples indicates that the modified assay displays a linearity of 0.98 and a least detectable dose of 1.3854 nmol/L. Intra- and interassay coefficients of variation were 3.91 and 5.26, respectively.

### Peer interaction playground paradigm

We designed an ecologically valid interaction that blends prescribed sequences of play while permitting considerable flexibility in the protocol to allow natural behavior to occur (see validity findings in Behavioral Coding below).

**Figure 2:**
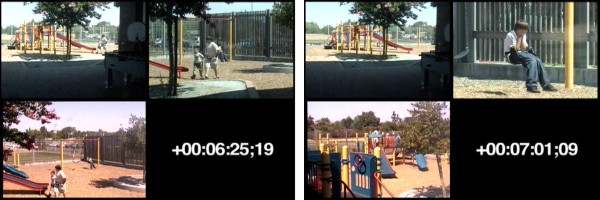
**Peer interaction playground video**. Three different points of view of the scene were recorded, synchronized into a uniform format and used to code the social transaction.

The paradigm occurs on a fenced 60" × 60" playground containing large equipment and open space for cooperative games [8,9]. In order not to impinge on the natural environment, research personnel remained in the building. The entire transaction was recorded with four cameras and sound equipment. Two fixed video cameras are housed on the building exterior in glass cases that rotate to observe different areas of the playground with one camera actively recording at any given time. Adjacent to the playground is a concealed video control room where the cameras are monitored and controlled. Two portable cameras were also used to record through windows (see Figure 2).

Thus, different points of view of the scene were recorded and synchronized into a uniform format using Apple Final Cut Pro 5 video editing software (Apple Computer Inc., Cupertino, CA, USA), resulting in a three-screen image used to code the social transaction (see Noldus below).

The interaction paradigm consists of a child with autism, a typically developing child, and an age-matched confederate. The confederate provides behavioral structure to the free play, permitting key interactive sequences that consistently occur within a natural social setting. Each child was provided with a fanny pack containing a small battery-powered microphone that was clipped to the child's shirt. Each device was recorded onto a separate channel on a sound mixer. The confederate wore earphones that enabled direct communication with research personnel who provided directive cues to ensure appropriate responding at key time points. The two participants were escorted to the playground simultaneously for a 20-minute play session that was divided into four 5-minute periods. The first period involves free play in which the three children engage in independent gross motor play. During the second period, the confederate solicits interaction on the play structures. The third period involves introduction of a box of toys (i.e., balls) that facilitate cooperative play. During the final period, the confederate solicits interaction surrounding one of the toys. Verbal cues at the 5-, 10- and 15-minute time periods were provided to the confederate via earphones to cue the confederate when to approach (second and fourth period) and when to engage in independent play (first and third period). If one or both of the child participants did not choose to play when asked, up to two additional bids were made spaced 1 minute apart. The confederate was fully trained and practiced on these basic procedures prior to the run of the study, and they were not told which child had autism or typical development.

### Behavioral coding

Traditional behavioral coding is not adequate to capture the dynamic socioemotional and regulatory factors proposed to be problematic in autism. Thus, to more fully describe the interplay between the children, we utilized the social transaction method to characterize the interchange as a whole, which has been used to carefully describe the complex social dynamics of nonhuman primates [36,37]. Several behavioral variables were used, including equipment use (noncooperative use of the surrounding environment for entertainment purposes), verbal rejections (verbalizations specifically intended to terminate or prevent interaction) and avoidant movements (deliberate changes in position to either exit or avoid an interaction). The percentage of a child's interactive participation was also examined.

The reliability and validity data for the peer interaction measure is very good. Coding of interrater reliability using Cohen's κ was K = 0.80, and test-retest reliability was 0.90 between two established raters. The peer interaction measure shows good face validity, and the construct validity is also strong. Specifically, as part of ongoing evaluation of the method, we compared the behavioral indices described below (e.g., percentage of time interacting, approach, avoid, gesture, equipment, proximity, reject) to a parent report questionnaire of social behavior in autism, the Social Responsiveness Scale (SRS) [47]. Using Pearson product correlations, we compared peer interaction variables during solicited motor (T2) and cooperative (T4) play. The correlation between the total SRS score and percentage of time interacting was *r *= -0.47 for T2 and *r *= -0.71 for T4, respectively, and the SRS subdomains had values ranging from (-0.38, -0.46) for T2 and (-0.63, -0.70) for T4. The results correlate in a predictable direction, with the standardized SRS demonstrating an association presumed related to aspects of social interaction. Furthermore, the correlations are moderate, suggesting that our peer interaction is measuring a new construct and not simply redundant with the SRS (e.g., *r *≥ 0.80), which would make the new construct unnecessary [49].

The Observer XT Version 8.0 (Noldus Information Technology, Leesburg, VA, USA) [50] was used for the collection and analysis of the interaction observational data. Analyses included (1) the standard ethological approach examining the frequency, duration and directionality of target behaviors and (2) the transactional approach or who-does-what-to-whom format with a predefined list of behaviors [51]. Specifically, we utilized a modification of the transactional method developed in studies of nonhuman primate social behavior [38,52]. In this method, social interactions are organized in bouts beginning with an overture by one individual, initiating a sequence of interactions between two or more participants. Bouts of interaction can be analyzed for their complexity (e.g., the number of distinct moves), duration, and for higher-order attributes, such as cooperation (interaction) and conflict (rejection).

The descriptive unit of a transactional bout identifies an actor's attempt to alter its immediate state of association with a target subject by means of either an affiliative (cooperative) or antagonistic (compete) response. Within each transactional episode, the immediate responses of the subjects and the responses of the other children were coded. Actions that initiate a bout included a hierarchy of responses such as spatial, verbal, nonverbal gestures and objects, among others. Shifts in orientation and proximity assisted in determining the end of the bout. Each participant's data was analyzed separately while simultaneously viewing all three video scenes to capture the optimal transaction moment.

## Data analysis

Independent two-sample *t*-tests were conducted to assess differences between the groups based on age and clinical variables. Previous results have shown IQ to differ significantly between groups; therefore, IQ was included in all behavior and cortisol models as a potential confounding variable if (1) it was found to independently predict the outcome variable and (2) the diagnosis coefficients changed by 10% or more. The behavioral variables avoidance, rejection, equipment use and interactions for time periods T2 and T4 (when interaction by the confederate was initiated) were analyzed using Poisson regression, with diagnosis and age included as main effects and interaction terms. Poisson regression models allow direct comparison of average rates of initiation, social rejection, and interaction between children with autism and typical children. The percentage of time spent interacting was treated as a continuous variable analyzed using standard linear regression and included the same set of independent variables and interactions used for the other behavior variables. Models were reduced using a backward selection method, removing the interaction term followed by main effects.

Salivary cortisol measurements are positive and skewed toward large values; thus, the log transformation was performed to achieve approximate normality and was used in all cortisol analyses. Baseline preplay cortisol measurements were compared to the average afternoon value using a paired *t*-test. Given that values might differ by diagnosis, baselines were compared to afternoon levels within each group. The stress response consisting of the four cortisol measurements (baseline, postplay, 20 minutes postplay, 40 minutes postplay) was characterized using a repeated-measures linear mixed-effects model treating the observations longitudinally. The model included time of measurement, age, SCQ and SRS as continuous main effects, diagnosis as a categorical main effect, and select two-way interactions. A backward selection method was used to reduce the model by first removing insignificant interactions followed by insignificant main effects. A Wald test was used to test the validity of the final model selected. A random effect was included per child, and error terms were assumed to be independent, normally distributed and to have a common variance.

The cortisol response was also dichotomized and used as an independent predictor of playground behavior. The maximum change in cortisol from arrival to either the 20-minute (S2) or 40-minute (S3) postbaseline measurements was calculated. We defined the 40% of participants with the highest cortisol response to stress as the cortisol responder group and the 40% with the lowest response as the cortisol nonresponder group [53]. An increase from baseline to the 20-minute period indicated a stress response to the beginning of the peer interaction, while an increase to the 40-minute period indicated a stress response to the end of the peer interaction. The behavioral variables were then reanalyzed using the methods described above with the additional main effect of responder status and select two-way interactions included.

## Results

Analyses were conducted between 21 children with autism and 24 neurotypical children. The means and standard deviations of the demographic and dependent variables are displayed in Table 1.

**Table 1:** Demographic and independent variables

	Autism			Neurotypical					
**Variable**	**Mean**	**SD**	**Range**	**Mean**	**SD**	**Range**	***t*-score**	***df***	***P *value**

Age	10.0	1.1	8.0-12.0	9.9	1.5	8.1-12.5	0.18	43	0.86
IQ	89.7	14.7	75-125	121.0	12.4	99-142	-7.77	43	< 0.0005
SRS	104.4	32.3	58-164	22.3	16.7	4-61	10.78	41	< 0.0005
SCQ	25.0	6.2	12-34	2.6	2.4	0-9	16.18	40	< 0.0005

SD, standard deviation; SRS, Social Responsiveness Scale; SCQ, Social Communication Questionnaire; *df *, degrees of freedom.

### Playground behavior

We were particularly interested in analyzing time periods during solicited motor (T2) and cooperative (T4) play when interaction by the confederate was initiated. The behavioral data generated with Noldus Observer XT [50], an observation and coding system (Noldus Information Technology, Leesburg, VA, USA (Table 2)), was analyzed using Poisson regression for count data, with diagnosis and age as main effects and interaction term. Diagnosis was a significant predictor of proximity during cooperative play (χ²(1) = 4.39, *P *= 0.036) such that children with autism more frequently entered and then left within close proximity of the other children without transitioning into an interaction. Analysis of initiations during cooperative play revealed diagnosis to be significant (χ²(1) = 4.03, *P *= 0.044), showing that typical children initiated more than children with autism. Children with autism were also found to reject significantly more (χ²(1) = 7.10, *P *= 0.008) during solicited play and displayed greater independent use of equipment (χ²(1) = 16.80, *P *< 0.0005) during cooperative play than neurotypical children. IQ was found to be an independent predictor of many of the behavior variables, including proximity and both independent and group equipment use; however, the diagnosis effect was not modified to a large degree in these models, indicating that IQ was not a significant confounding variable.

**Table 2:** Peer interaction behavioral means and standard deviations by diagnosis

	Solicited motor play (T2)		Cooperative play (T4)	
	
	Autism	Neurotypical	Autism	Neurotypical
Variable	Mean (SD)	Mean (SD)	Mean (SD)	Mean (SD)
Approach	0.86 (1.24)	0.63 (0.97)	0.57 (0.75)	0.63 (0.92)
Gesture	0.48 (0.68)	0.50 (1.18)	0.29 (0.56)	0.75 (1.54)
Equipment use	2.48 (2.46)	1.75 (2.13)	1.89 (2.13)	0.46 (0.66)
Equipment use - gGroup	2.19 (2.52)	3.63 (3.32)	2.19 (2.14)	2.63 (1.58)
Proximity	0.38 (0.74)	0.63 (1.31)	0.43 (0.81)	0.08 (0.28)
Initiate	0.65 (0.93)	0.36 (0.73)	0.12 (0.33)	0.54 (0.86)
Reject	0.82 (1.01)	0.18 (0.39)	0.88 (1.11)	0.41 (0.96)
% time interacting	46 (31)	79 (24)	59 (39)	91 (13)

SD, standard deviation.

**Figure 3:**
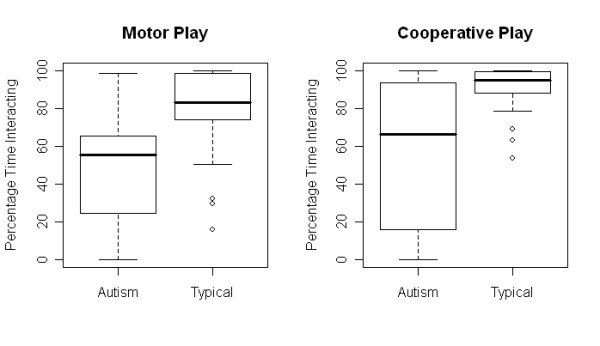
**Boxplots of diagnosis and social interaction**. Solicited motor play and cooperative play.

The percentage of total time interacting was treated as a continuous variable and analyzed using linear regression. Children with autism engaged in less time interacting during both motor (F(1,43) = 16.7, *P *= 0.0002) and cooperative play (F(1,43) = 14.78, *P *= 0.0004) (see Figure 3). These data lend support for our hypothesis showing less interaction during natural play for the children with autism.

### Cortisol responsivity

Comparison of the baseline cortisol measurement to the average afternoon level found a significant difference for the typical children (t(22) = 2.23, *P *= 0.04), indicating a higher than expected cortisol level at the beginning of the peer interaction. However, two of the younger typical children had extreme outlying values (>2 standard deviations from the mean), and when removed, the differences were no longer statistically significant (t(20) = 1.54, *P *= 0.14). Compared with their own home values, the children with autism did not have significantly different cortisol levels (t(19) = 0.54, *P *= 0.60).

**Figure 4:**
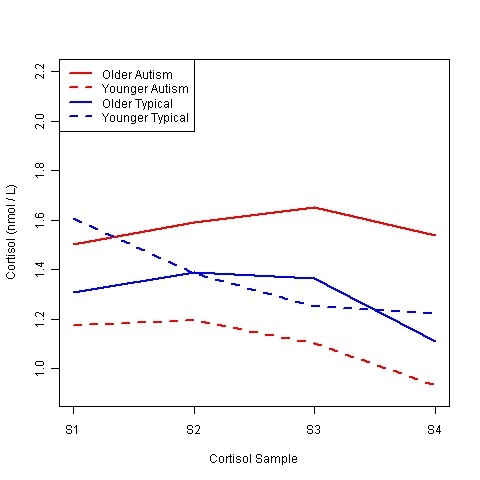
**Group patterns of average cortisol response to the peer interaction**. Figure age subgroups (Older, Younger) were separated by median age within each diagnostic group. S1 = Baseline (Preplay), S2 = Postplay, S3 = 20 minutes postplay, S4 = 40 minutes postplay. nmol/L = nanomoles per liter.

The repeated-measures analysis of the cortisol values revealed a significant model (χ²(4) = 22.76, *P *< 0.0005) that included time, diagnosis and age as main effects and a diagnosis × age interaction (see Table 3). The SRS and SCQ main effects were not significant (*P *> 0.05). In addition, the interaction terms between time and either age or diagnosis were not significant (all *P *> 0.05). Therefore, in the absence of interaction, the time main effect can be interpreted directly such that higher cortisol levels occurred at baseline, with roughly a 5% decrease for every additional 20-minute time period. Since the values in the laboratory were similar to the afternoon values at home, we interpret this as the normal circadian decline expected in the afternoon. The significant diagnosis × age interaction suggests that age is an important predictor of cortisol for children with autism such that older children experience higher cortisol levels across time. Although age was treated as a continuous variable, Figure 4 displays age based on a median split (younger vs. older), which was 9.8 years for both groups. Cortisol levels for typical children, however, were not affected at a statistically significant level by age. IQ was not found to be a significant independent predictor of cortisol levels and therefore was not included in the models as a confounding variable.

**Table 3:** Model coefficients for the cortisol stress response

Variable	Estimate (SE)
Intercept	1.457 (0.490)
Time	-0.004 (0.001)
Diagnosis	-2.371 (0.933)
Age	-0.001 (0.049)
Diagnosis* age	0.238 (0.095)
Number of observations per participant	4
Standard deviation of random effect	0.277

SE, standard error of estimate.

The playground behavior variables were analyzed again with responder status included with the other independent variables. Analysis of avoidance during cooperative play yielded a model with both age and responder status as main effects (χ²(2) = 9.25, *P *= 0.009), showing that cortisol responders avoided at a higher average rate than nonresponders and younger children had a greater avoidance rate than older children. Responder status was also a significant predictor of gesturing during both motor (χ²(1) = 5.52, *P *= 0.018) and cooperative play (χ²(1) = 4.53, *P *= 0.033) such that children exhibiting a heightened cortisol response gestured less often than those classified as nonresponders.

More complex models that included responder status, age and their interaction were found that explained group equipment use for both solicited motor play (χ²(3) = 12.3, *P *= 0.006) and cooperative play (χ²(3) = 8.24, *P *= 0.04) where older children who were stress responders had the highest rates of equipment use with others.

## Discussion

The investigation was conducted to evaluate the behavioral and physiological response to social interaction on a playground with novel peers. As predicted, the children with autism spent less time interacting during free play and solicited cooperative play (see Figure 4), which is similar to previous findings of social interactions during unstructured social activities in autism [5]. The children with autism exhibited more rejection during solicited play and tended to play more independently with the equipment than their typically developing peers. Importantly, however, the children with autism did engage in interaction to varying degrees and tended to respond to rather than initiate the play.

In regard to cortisol responsivity related to playground exposure, a statistical interaction was observed on the basis of diagnosis and age. Specifically, within the autism group, children experienced higher cortisol levels with increasing age, while among typical children cortisol levels were not modified by age. These findings suggest that some children with autism evidenced an enhanced HPA response during social play leading to elevated cortisol.

At first glance, it may be interpreted that socialization, at least with peers, is stressful and may be deemed threatening in some way. Interestingly, however, many of the children with autism did not completely remove themselves from the interaction as one might expect from a threatening situation. Older children with autism tended to play more in a group on the equipment during both motor and cooperative play and avoided less, indicating that they were voluntarily engaged in the interaction. The younger children tended to avoid interacting at a higher rate than the older children with autism. Thus, taken together, it may be that children with autism responded to the playground situation with an approach/avoidance conflict; they wanted to play but found it possibility threatening. We conjecture that younger children with autism responded to the conflict with greater avoidance, while older children with autism yielded to the attraction in spite of concurrent LHPA activation. Meanwhile, the neurotypical children did not perceive the playground interaction as threatening, which is reflected in their biobehavioral profile.

Developmental changes in the HPA axis have been shown in nonhuman primates [54], children [55] and adolescents [56]. It is important to note that in neurotypical children, blunted cortisol responsivity to social stress has been reported in children 11 and 13 years of age compared to younger 9-year-old children [56], which is a sharp contrast to older children with autism. The inverse relationship in typically developing children suggests that the difference is not simply attributed to developmental factors. Since older children with autism show enhanced cortisol with social engagement, the comparison suggests that there is something unique about this developmental stage and social stress in autism. Importantly, since we enrolled only prepubertal children, the results do not appear to be associated with puberty.

Interestingly, the parent report measures of social functioning (SRS and SCQ) were not predictive of cortisol responsiveness or social interaction patterns on the playground for either group. These measures are broad-based and less refined in being able to discern real-time social interaction which contributed to the need to develop the peer interaction paradigm. Although parent report measures are good at distinguishing children with autism from neurotypical children or identifying global problems, they are less able to identify discrete behaviors that may map onto neuroendocrine profiles.

It has long been shown that social variables can induce, enhance or diminish the stress response in primates [34]. Increased cortisol may not always overlap onto fear-based constructs. It has been suggested that cortisol levels in inhibited children may be associated with poor coping strategies rather than being related to threat or fear [57]. In this way, elevations in cortisol could be the result of a failure to have adaptive responses or coping strategies to appropriately respond in the context. It has been suggested that awareness of such limitations may increase with age, contributing to enhanced social and evaluative anxiety in individuals with ASD [22,58]. Importantly, the stress response does not inhibit the older children from at least partially engaging in the interaction which is a stressful but potentially rewarding situation.

Therefore, the findings may be interpreted within the context of social competency such that it is not merely a matter of the social situation being stressful; rather, the competency to engage in the interaction plays a role in determining the magnitude of the LHPA response. It is likely that the enhanced cortisol levels in the older children with autism reflect preparation for social interaction amid greater awareness of their own social limitations. In a study of young children, elevations in cortisol were associated with age and greater approach behavior to the new peer situation [33]. Teaching coping strategies along with social skills may go far in improving social competence while ameliorating the increased reactivity to novel social situations.

It was previously suggested that social anxiety in ASD resulted from repeated failure in social interaction [59], which could also be associated with enhanced responsivity of the LHPA axis. Recent reports suggest that as some youth with autism age, they gain greater insight into their social impairment, which leads to increased anxiety [22,58,59]. It may be reasoned that the older child characterized by more social interactions, increased self-awareness and higher biological and psychological stress would likely benefit from treatment comprised of direct social skills training, stress reduction techniques and safe opportunities to interact and practice skills with others [60]. Conversely, younger children characterized by withdrawn behavior, reduced motivation and lower stress may benefit from treatment approaches that utilize video or computer media and less reliance on face-to-face interaction to acquire skills [40]. They may also be taught to endure or cope with the stress in support of the pleasure that comes from social interaction. It is unclear if the limited social engagement is due to a lack of social interest or an attempt to limit exposure to situations that are potentially threatening. The clinical relevance of the findings may indicate an important developmental shift in which children with autism begin to engage in less social avoidance that is also coupled with increased physiological arousal. Additional studies that include longitudinal designs are needed to disentangle these age- or developmentally related factors.

What remains unclear from these data is which neuroendocrine pathways or brain structures may be implicated? A likely candidate is the amygdala, which is a key regulator of the HPA axis and has been implicated in the neuropathology of autism in regard to both structure and function (e.g., [25,61-65]), which has been further associated with individual factors of age [64] and anxiety in individuals with autism [62,66]. It is also apparent that other neurohormones, including oxytocin, which have been associated with autism [67-71] and shown to moderate stress responsivity may play a role in the phenotypic social stress profile in autism.

The playground interaction described herein involves only three children and includes solicited but not forced interaction. The fact that the children with autism exhibited increased stress under such benign social conditions is concerning. Social stress may be even more significant under conventional school playground environments with more children, increased stimulation and more rejecting social exchanges.

The study includes a new peer interaction paradigm designed to emulate a "real life" playground environment. Regarding potential limitations, it may be that the novelty of the situation was the critical component as opposed to the social aspects per se. However, the association between responder status and social engagement tends to refute this notion. A study regarding introduction to a play setting without peers could disentangle this potential confound. The design also included a brief adaptation period of approximately 15 minutes prior to going out on the playground. Some may argue that this period was insufficient to allow participants to fully adapt to the setting and recover from anticipatory elevations [41]. The time period was a compromise, as an extended period of time might result in frustration. Importantly, the children adapted in a private, nonclinical room with couches and tables to elicit calmness before and after the peer interaction.

## Conclusions

In summary, the data provide strong support for our behavioral coding method in being able to distinguish between children with autism and neurotypical children, as well as being able to identify individual differences within the autism group. In response to the natural peer interaction paradigm, distinct patterns emerged within the autism group on the basis of developmental, biological and behavioral response patterns. Taken together, these data suggest that many, but not all, children with autism mount measurable stress responses in relatively benign social situations and that these appear to be a function of age and level of social engagement.

## Competing interests

The authors declare that they have no competing interests.

## Authors' contributions

BAC conceived of the study, developed the peer interaction paradigm with SPM, conducted and supervised the clinical evaluations, and drafted the initial manuscript. CWS participated in the design of the study, conducted the statistical analysis and prepared the results and statistical figures for publication. DS participated in the development of the peer interaction coding scheme, conducted and supervised the coding and coordinated the study. NR participated in study design, administered the protocols, collected salivary samples, and participated in manuscript preparation. SPM developed the peer interaction paradigm with BAC, supervised the cortisol assays, engaged in the interpretation of the results and contributed to the manuscript. All authors read and approved the final manuscript.

## Consent

Written informed consent was obtained from the patients for publication of this manuscript and accompanying images. A copy of the written consent is available for review by the Editor-in-Chief of this journal.
